# A Preliminary Investigation of the Effects of a Western Diet on Hippocampal Volume in Children

**DOI:** 10.3389/fped.2020.00058

**Published:** 2020-03-03

**Authors:** Jill Stadterman, Kyrstin Belthoff, Ying Han, Amanda D. Kadesh, Yuliya Yoncheva, Amy Krain Roy

**Affiliations:** ^1^Pediatric Emotion Regulation Lab, Department of Psychology, Fordham University, The Bronx, NY, United States; ^2^Department of Child and Adolescent Psychiatry, Hassenfeld Children's Hospital at New York University Langone Health, New York, NY, United States

**Keywords:** western diet (WD), dietary fat, hippocampus, neuroimaging (anatomical), children

## Abstract

**Introduction:** Over the course of the 20th century, there has been a sharp increase in the consumption of saturated fat and refined sugars. This so-called “western diet” (WD) has been extensively linked to biological alterations and associated functional deficits in the hippocampus of animals. However, the effects of a WD on the human hippocampus are less well-characterized. This preliminary study aimed to extend prior animal work by investigating the effects of a WD on hippocampal volume in children.

**Methods:** Twenty-one healthy children (ages 5–9) completed a structural T1-weighted magnetic resonance imaging scan. Bilateral hippocampal volumes (as regions-of-interest) and bilateral amygdala volumes (as medial temporal lobe control regions-of-interest) were calculated. WD variables were derived from the parent-completed Youth/Adolescent Food Frequency Questionnaire. Specifically, variables were calculated as percent of daily calories consumed from sugars, fats, or a combination of these (WD).

**Results:** While the relationships between overall WD consumption and bilateral hippocampal volumes were not significant, increased fat consumption was significantly related to decreased left hippocampal volume. Sugar consumption was not related to hippocampal size. Control region volumes were not related to any diet variables.

**Discussion:** This study is the first to directly link diet—specifically fat consumption—to decreased left hippocampal volume in children. This extends previous work showing smaller left hippocampal volume related to obesity in pediatric samples. Though preliminary, findings represent an important step toward understanding the impact of diet on child brain development.

## Introduction

Over approximately the last 10,000 years, the dietary pattern of humans has shifted from a hunter-gatherer approach to the utilization of agriculture and animal farming (1). With this shift, there has been a sharp increase in the consumption of fats and refined sugar and a simultaneous decrease in fiber and vegetable intake (1). This so-called “western diet” (WD) is composed of foods that include high-fat meats, dairy, cereal, refined grains, and ultra-processed foods, such as fast food and foods made with high fructose corn-syrup. Consumption of a WD is commonplace in the United States (1). For example, more than 60% of Americans consume fat and sugar in excess of the amounts recommended by United States Dietary Guidelines (2). This overconsumption of fat and sugar, which has occurred too quickly on an evolutionary timescale for our genome to adapt (1), is posited to be a major factor in many modern medical problems, such as obesity, hypertension, and diabetes (3). A WD has also been linked to brain functioning, including global cognitive deterioration (4, 5), while a Mediterranean diet, or a diet characterized by high consumption of olive oil and plant-based foods, has been shown be a protective factor against normal cognitive aging (6) as well as Alzheimer's Disease (7). Taken together, this body of work suggests that diet can substantially impact the body and the brain.

While many brain structures and functions may be affected by a WD, extensive animal research points specifically to its impact on the hippocampus. Responsible for encoding and reconsolidation phases of long-term memory as well as spatial memory, the hippocampus appears to be particularly susceptible to neurotoxic damage, and this is possibly because it is one of only three areas in the brain where neurogenesis continues to occur throughout the lifespan (8). Animal work, primarily in adult rodents, has demonstrated that diets high in fat and sugar both increase neurodegeneration (9) and decrease hippocampal neurogenesis (10, 11). For example, rats fed a WD for 2 years exhibited significantly decreased hippocampal brain-derived neurotropic factor (BDNF), a protein that supports neuron growth, compared to rats fed a control diet (12). While this result was seen after a substantial amount of time on a high-fat diet, other work has shown significantly reduced hippocampal microtubule associated protein 2 (MAP-2), a protein responsible for stabilizing dendrites during neuron growth, after just 8 weeks on a high-fat diet (13, 14). The decreases in both BDNF and MAP-2 were also correlated with hippocampal-dependent memory deficits.

WD consumption has also been shown to increase the permeability of the hippocampal blood–brain barrier (BBB). One study found that rats fed a WD for 3 months had a significantly decreased mRNA expression of tight junction proteins in the hippocampus, an indicator of increased hippocampal BBB permeability, compared to rats fed a control diet (15). Rats in the WD condition also had impairments on non-spatial hippocampal-dependent memory tasks. Additional work has found similar results in adult rats fed a high-fat, high-cholesterol diet (16) as well as in obese rats (17, 18). In humans, elevated BMI has been shown to predict increased BBB permeability at 24-years follow up (19). Overall, while there is significant evidence of an impact of diet on hippocampal volume in animal models, it is important to note that individual differences may impact this association. For example, Trevino et al. (9) observed that only male rodents, not female rodents, had decreased hippocampal neurogenesis after consumption of a high-fat, high-sugar diet. Further, evidence suggests that diet-induced increases in permeability of the hippocampal BBB may only be present in rats who developed obesity from WD consumption and not their normal-weight counterparts (17, 18).

While most animal studies use adult rats, there is a subset of literature suggesting that the effects of WD consumption on the hippocampus may be particularly salient during adolescence. Specifically, one study found that adolescent rats, but not young adult rats, demonstrated impairment on a hippocampal-dependent memory task after exposure to a high-fat diet (20). Similar results have been found for adolescent rats fed a high-sugar diet (21), with one recent study demonstrating that these memory deficits persist into adulthood (22). Biological alterations, such as decreased hippocampal neurogenesis and increased concentration of proinflammatory cytokines in the hippocampus, have also been found after WD exposure in adolescent rats but not their adult counterparts (21, 23).

Despite robust animal work suggesting both functional and structural hippocampal alterations after WD exposure, investigations of this relationship in humans is sparse. Recent evidence suggests that increased WD intake is associated with poorer performance on tasks relating to visual-spatial and long-term memory in a large sample of adolescents (24). Moreover, increased consumption of saturated fatty acids is associated with deficits in both hippocampal-dependent and hippocampal-independent memory tasks in children [ages 7–9; (25)], and higher levels of dietary fat and sugar are associated with decreased performance during hippocampal-dependent memory tasks in adults (26–28). In regard to hippocampal volume, a longitudinal study of older adults found that individuals who consumed a WD had significantly decreased left hippocampal volume at 4-years follow-up as compared to those with a more prudent, Mediterranean-style diet (29). Another recent longitudinal study of adults similarly found that individuals who scored higher on a diet index (suggesting a decreased risk for chronic diseases) had larger hippocampal volumes 13 years later when compared to adults scoring lower on the index (30). Although this relationship was seen in both hemispheres, it was stronger for the left hippocampus. Furthermore, similar reductions in left hippocampal volume have been found in obese children compared to healthy weight peers [ages 6–8 and 8–12, respectively, (31, 32)]. However, as multiple factors can contribute to obesity, the specific impact of diet on hippocampal volume in early development remains unknown.

The present study aimed to extend prior work by conducting a preliminary examination of the relationship between consumption of a WD and bilateral hippocampal volumes in children ages 5–9. Using a diet questionnaire completed by children's parents and hippocampal volumes obtained from anatomical MRI scans, we investigated the relationship between WD consumption and hippocampal size. Based on prior studies, we hypothesized that higher fat and sugar consumption would be correlated with decreased hippocampal volume, particularly in the left hemisphere (29–32). We further examined the individual contributions of sugar and fat consumption to hippocampal volume differences. We repeated these analyses using the bilateral amygdala as a medial temporal lobe control region-of-interest.

## Method

### Participants

Participants were typically developing boys and girls (ages 5–9 years) recruited as part of a study of children with disruptive behavior disorders. Participants were required to be free of any DSM-IV-TR diagnosis (33), other than enuresis, to speak English, and to have an estimated IQ of >75, as measured by the Kaufman Brief Intelligence Test, Second Edition [KBIT-2; (34)]. Exclusions for MRI contraindications included the presence of metal in the body or a history of a head injury or neurological disorder. A total of 51 typically developing children (TDC) participated in the larger study. The current analyses only included the 21 children who successfully completed an anatomical MRI scan and whose parent completed the Youth/Adolescent Food Frequency Questionnaire [YAQ, described below; (35)]. Parents also completed a comprehensive demographics form in which average annual income was recorded.

### Study Procedures

The study was conducted at Fordham University and the New York University School of Medicine. Both institutional review boards approved the study. During the initial evaluation visit, written parental consent and child verbal (ages 5–6) or written (ages 7–9) assent were obtained. Children then completed several clinical assessments including the KBIT-2 and an MRI habituation session in which they practiced keeping still in a scanner stimulator while watching a movie and receiving behavioral feedback on how much they moved. Parents were administered the Kiddie Schedule for Affective Disorders and Schizophrenia for School-Age Children-Present and Lifetime Version [K-SADS-PL; (36)] by either a post-doctoral fellow or clinical psychology doctoral student. Absence of clinical diagnoses was confirmed in a weekly consensus meeting with the study team and the study PI, a licensed psychologist. Parents also completed the Edinburgh Handedness Inventory [EDI; (37)] as well as the Youth/Adolescent Food Frequency Questionnaire [YAQ; (35)], a 193-item measure that asks about dietary patterns over the last year. Consumption of specific food items is generally rated from “never/not in the last month” to “1 or more times a day”/ “more than 4 times per week,” although exact rating options vary slightly by food item. Responses were then converted into estimated daily consumption of various nutritional variables, including total calories, total fat, and total sugar, which were the primary variables of interest in this study. Participant weight was also obtained via self-report, either from the YAQ questionnaire, or for families who did not complete that item, from the MRI screening form completed by the parent at the time of the MRI scan. To control for differences in weight associated with participant age and sex, age- and sex-based weight percentiles based on the Centers for Disease Control charts (38, 39) were used in the study analyses.

Approximately 1–2 weeks following the initial session, participants completed an MRI scan at the NYU Center for Brain Imaging using a 3T Siemens Allegra scanner with a single-channel Nova head coil. Anatomical T1-weighted images were obtained using 3D Magnetization Prepared Rapid Acquisition Gradient Echo sequence (TR = 2,530 ms; TE = 3.25 ms; TI = 1,100 ms; flip angle = 7°, 1 mm^3^ isotropic voxels). Functional MRI scans were also obtained, but results are not reported here.

### Data Analyses

Structural T1-weighted images were analyzed using the standard fully-automated, validated segmentation algorithms implemented in FreeSurfer version 5.3.0^1^ (40, 41) following the established protocol (42) detailed in the online imaging protocols^2^. Following image preprocessing using the “recon-all” pipeline^3^, visual inspection of each subject's segmentation was carried out for quality assurance. Left and right hippocampal and amygdala volumes, along with estimated total intracranial volume were calculated and analyzed.

Western diet (WD) was calculated by converting raw values of the amount of total fat (sum of animal fat and vegetable fat) and total sugar (sum of lactose, fructose, glucose, maltose, and sucrose) consumed based on the FFQ into percentage of daily calories. First, grams of fat were multiplied by nine (43) and grams of sugar were multiplied by four (44) to derive daily calories from fat and sugar, respectively. Then, these values were subsequently divided by total daily calories consumed according to the FFQ, which resulted in percentage of daily calories from fat and percentage of daily calories from sugar. These two variables were summed to get the WD variable.

Initial analyses were conducted to assess for normality and outliers in the primary variables including bilateral hippocampal and amygdala volumes, daily calories from fat, daily calories from sugar, and WD. One participant was identified as an outlier (two standard deviations from the mean) and excluded from the daily sugar analysis. No other outliers were detected. In light of previous work demonstrating an association between socioeconomic status (SES) and hippocampal volume (45) as well as BMI and hippocampal volume (31, 32), we first used correlation analyses to assess the presence of a relationship between SES, operationalized as annual family income, and weight percentile with left and right hippocampal volumes, respectively. Primary study hypotheses regarding associations between diet variables and hippocampal volume were then tested using multiple regression analyses, covarying for total intracranial volume. Separate regressions were conducted for left and right hippocampal volumes. These regression analyses for each diet variable were repeated separately for left and right amygdala (control regions) volumes.

## Results

Participants included 21 children aged 5–9 (*M* = 7.4; *SD* = 1.4; 81% male), and 52% of participants identified as White and 23.8% as Hispanic. Eighteen participants (86%) were right handed and three (14%) were ambidextrous, according to the EDI. One participant had a diagnosis of enuresis. Full demographic characteristics are presented in Table 1. On average, WD accounted for 54.8% (*SD* = 0.04) of participants' daily calorie consumption; 23.6% (*SD* = 0.04) of daily intake was from sugar and 31.1% (*SD* = 0.04) from fat. Descriptive statistics of the components of fat and sugar are presented in Table 2. Descriptive statistics of bilateral hippocampal and amygdala volumes are presented in Table 3.

**Table 1:** Demographic information (*N* = 21).

**Demographic information**	**Count**
Mean age in years (*SD*)	7.4 (1.4)
Males (%)	17 (81)
**Handedness**	
Right (%)	18 (86)
Ambidextrous	3 (14%)
**Race**	
White or Caucasian (%)	11 (52)
Black or African American (%)	3 (14)
Asian or Pacific Islander (%)	2 (10)
Other/Mixed (%)	5 (24)
**Ethnicity**	
Hispanic (%)	5 (24)
**Annual Family Income**	
$34,999 or less (%)	2 (10)
$35,000–$99,000 (%)	5 (24)
$100,000 or more (%)	13 (62)
Missing (%)	1 (5)
**Mean Weight Percentile (*****SD*****)**	59.0 (28.3)
0–25th percentile (%)	3 (14)
25–50th percentile (%)	4 (19)
51–75th percentile (%)	5 (24)
75th percentile and above (%)	9 (43)

**Table 2:** Components of diet variables.

**WD Components**	**Mean in grams**
**Total Fat**	**78.19**
Animal fat	37.78 (48.3%)
Vegetable fat	40.42 (51.7%)
**Total Sugar**	**135.60**
Fructose	32.46 (23.9%)
Sucrose	49.77 (36.7%)
Maltose	3.31 (2.4%)
Glucose	28.05 (20.7%)
Lactose	22.00 (16.2%)

**Table 3:** Mean hippocampal and amygdala volumes (mm^3^).

**Brian Region**	**Mean *(SD)***
Left hippocampus	3736.17 (348.0)
Right hippocampus	3763.76 (434.6)
Left amygdala	1381.37 (193.0)
Right amygdala	1564.16 (194.2)

No significant associations were found between SES and hippocampal volume nor between weight percentile and hippocampal volume. Tests of primary hypotheses found small, non-significant associations between percentage of daily calories from a WD and both right and left hippocampal volume when controlling for total intracranial volume [left hippocampus: partial *r* = −0.16, *F*_(1,18)_ = 0.49, $R_{Change}^{2}$ = 0.03, *p* > 0.05; right hippocampus: partial *r* = 0.06, *F*_(1,18)_ = 0.07, $R_{Change}^{2}$ = 0.00, *p* > 0.05].

**Figure 1:**
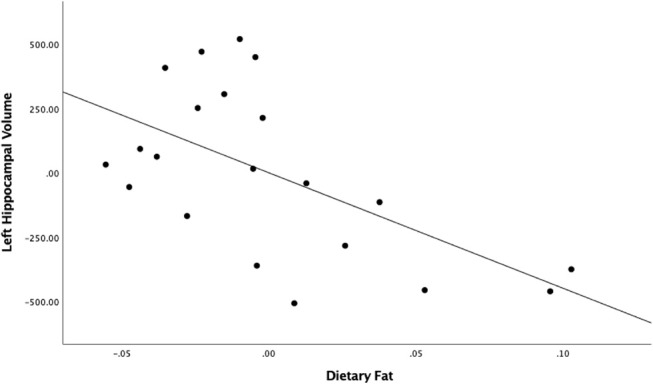
Unstandardized residuals of dietary fat and left hippocampal volume after controlling for total intracranial volume.

When evaluating the roles of fat and sugar separately, the regression model between fat and left hippocampal volume found a large and significant relationship [partial *r* = −0.57, *F*_(1,18)_ = 9.57, $R_{Change}^{2}$ = 0.33, *p* = 0.0063; Figure 1]. This result survived the Bonferroni correction for multiple comparisons (46). The relationship between fat and the right hippocampal volume was not statistically significant [partial *r* = −0.21, *F*_(1,18)_ = 0.85, $R_{Change}^{2}$ = 0.04, *p* > 0.05]. The relationships between sugar intake and both the left and right hippocampal volumes also failed to reach significance [left hippocampus: partial *r* = 0.27, *F*_(1,17)_ = 1.56, $R_{Change}^{2}$ = 0.07, *p* > 0.05; right hippocampus: partial *r* = 0.09, *F*_(1,17)_ = 0.16, $R_{Change}^{2}$ = 0.01, *p* > 0.05]. As expected, no significant associations were found between any diet variables and bilateral amygdala volumes.

## Discussion

The aim of this preliminary study was to extend previous animal and human findings by investigating the relationship between WD intake and hippocampal volume in children. While results did not support a significant effect of WD intake on hippocampal volume, greater fat intake was related to decreased left hippocampal volume—a finding that is in line with two prior adult studies (29, 30). Studies in animals have also found similar results, including impaired neurogenesis (10, 11) and increased neurodegeneration (9) in the hippocampus following WD consumption. Previous work with children has found a relationship between obesity and left hippocampal volume (31, 32). However, these studies did not investigate diet specifically. As such, our findings are the first to directly link diet, specifically fat consumption, to left hippocampal volume in children. This study adds to the growing body of animal and human literature demonstrating that diet affects the brain (47–49), and the hippocampus specifically, across the lifespan (15, 21, 23, 29, 30, 50).

We found an association between hippocampal size and daily fat consumption but not daily sugar or overall WD. While previous investigations have found WD consumption predicted smaller hippocampal volume after longitudinal follow-up (29, 30), neither finding was specific to fat. It is possible that, in both studies, fat was driving the association between WD intake and hippocampal volume, but because individual components of diet were not directly assessed, this association is unknown. This could explain the discrepancy among our findings. Further, prior animal work has found that increased fat intake over 6 months alters the vascular structure of the hippocampus (16), suggesting that changes in blood flow from excess daily fat consumption may be responsible for structural alterations to the hippocampus.

Moreover, as in line with prior work, our finding was localized to the left hemisphere. WD was not significantly associated with the right hippocampus or bilateral amygdala volume, suggesting that the left hippocampus may be particularly susceptible to diet-induced alterations, and to neurodegeneration more generally. A meta-analysis investigating hippocampal changes in Alzheimer's Disease and Mild Cognitive Impairment found that, while hippocampal atrophy was seen bilaterally, the effect was more prominent on the left side in both conditions (51). Furthermore, another study compared the effects of Alzheimer's Disease across hemispheres, finding increased vascular lesions in left hemisphere, although the authors hypothesize this may be part of normal aging (52). Taken together, these studies provide preliminary evidence that the left hippocampus may be uniquely susceptible to neurodegeneration. Further work is needed to understand the possible differential impact of environmental influences such as diet on the left hippocampus as compared to the right.

While these preliminary results are intriguing, this study is not without limitations; most notably, the study had a small sample size. It is possible that we did not have the power to detect small but significant effects, indicating a larger sample size is needed. Second, the use of food frequency questionnaires to quantify diet has been criticized in light of measurement error (53). For example, test-retest correlations of YAQ-quantified dietary variables measured at baseline and 1-year follow-up hover around 0.5 (54). Similarly, correlations comparing YAQ-quantified diet variables to daily dietary recall are also around 0.5 (55). However, despite the moderate reliability and validity of this measure, the YAQ, along with many other food frequency questionnaires, remain the primary method used to quantify diet in research (53), although improvement of these measures represents an important area for future work. Moreover, both animal and human studies suggest that WD exposure impacts hippocampal function, assessed behaviorally (15, 28, 56). We were unable to look at hippocampal memory impairment, as hippocampal-sensitive memory measures were not obtained. However, given the animal literature linking biological alterations and functional deficits (12, 15), we predict that we would see similar findings in humans. To our knowledge, no human work to date has investigated both hippocampal structural and functional alterations simultaneously, and, as such, this is an important next step. Lastly, in this study, WD was conceptualized dimensionally; future studies may consider investigating WD as a categorical variable, comparing hippocampal size between children who consume a WD and children who consume other style diets (e.g., vegetarian or Mediterranean) or children living in other parts of the world, where a WD may not be as common.

In conclusion, this study builds upon previous findings, showing a significant relationship between dietary fat intake and decreased left hippocampal volume. The impact of this work lies in its translational nature, as it strengthens the bridge from animal findings to human studies and provides convincing preliminary evidence for future investigation of the relationship between WD consumption and hippocampal functioning.

## Data Availability Statement

The datasets generated for this study are available on request to the corresponding author.

## Ethics Statement

The studies involving human participants were reviewed and approved by Fordham University Institutional Review Board, New York University School of Medicine Institutional Review Board. Written informed consent to participate in this study was provided by the participants' legal guardian/next of kin.

## Author Contributions

JS conducted statistical analyses and contributed to manuscript composition. KB contributed to manuscript composition. YH conducted statistical analyses. AK contributed to data collection and study design. YY contributed to MRI data collection and neuroimaging analyses. AR oversaw all aspects of the study.

### Conflict of Interest

The authors declare that the research was conducted in the absence of any commercial or financial relationships that could be construed as a potential conflict of interest.

## Footnotes

^1^http://surfer.nmr.mgh.harvard.edu/

^2^http://enigma.ini.usc.edu/protocols/imaging-protocols/

^3^http://surfer.nmr.mgh.harvard.edu/fswiki/recon-all
